# Acute Endocrine Responses to Different Strength Exercise Order in Men

**DOI:** 10.2478/hukin-2014-0116

**Published:** 2014-12-30

**Authors:** Rodrigo Rodrigues da Conceição, Roberto Simão, Anderson Luiz B. Silveira, Gabriel Costa e Silva, Marcelo Nobre, Veronica P. Salerno, Jefferson Novaes

**Affiliations:** 1 Department of Physiological Sciences and, Department of Physical Education and Sports, Federal Rural University of Rio de Janeiro - UFRRJ - Rio de Janeiro, RJ, Brazil.; 2 School of Sports and Physical Education, Rio de Janeiro Federal University - UFRJ - Rio de Janeiro, RJ, Brazil.

**Keywords:** strength training, hormones, physical fitness, athletic performance

## Abstract

This study compared the effects of order of muscle groups’ exercised (larger to smaller muscles vs. smaller to larger muscles) on the acute levels of total testosterone, free testosterone and cortisol during resistance training (RT) sessions. Healthy male participants (n=8; age: 28.8 ± 6.4 years; body mass: 87.0 ± 10.6 kg; body height: 181.0 ± 0.7 cm; BMI: 26.5 ± 4.1) were randomly separated into two experimental groups. The first group (LG-SM) performed an RT session (3 sets of 10 repetitions and a 2 min rest period) of the exercises in following order: bench press (BP), lat pulldown (LP), barbell shoulder press (BSP), triceps pushdown (TP) and barbell cut (BC). The second group (SM-LG) performed an RT session in following order: BC, TP, BSP, LA, BP. Blood was collected at the end of the last repetition of each session. Control samples of blood were taken after 30 min of rest. Significant differences were observed in the concentrations of total testosterone (p < 0.05), free testosterone (p < 0.0001) and cortisol (p < 0.0001) after both RT sessions in comparison to rest. However, when comparing LG-SM and SM-LG, no significant differences were found. The results suggest that, while RT sessions induce an acute change in the levels of testosterone and cortisol, this response is independent of the order of exercising muscle groups.

## Introduction

According to the American College of Sports Medicine (ACSM, 2009), the main methodological prescription variables are a load, volume, rest interval between sets/exercises, frequency of sessions, exercise modality, repetition velocity and exercise order. Among these variables, exercise order has been studied less frequently in controlled investigations. Current guidelines for the resistance training (RT) program design recommend that large muscle-group exercises generally should be performed first in a training session (ACSM, 2009; Fleck and Kraemer, 2004).

In a recent review, Simão et al. (2012) showed that exercise order affected repetition performance over multiple sets, indicating that total volume (number of repetitions) was greater when an exercise was placed at the beginning of an RT session, regardless of the relative amount of muscle mass involved. Exercises performed at the end of the session were associated with less repetitions, regardless of whether the movement involved a small muscle group, as in single-joint exercises, or a large muscle group, as in multijoint exercises. In terms of chronic adaptations, greater increases in strength were noted among untrained subjects for the first exercise of a given sequence, while lesser increases in strength were measured for the last exercise of a given sequence. Additionally, based on effect-size data for strength and hypertrophy, research suggests that exercise order should be arranged in priority of importance according to the training program’s goal, and irrespective of whether the exercise involves a relatively large or small muscle group (Dias et al., 2010; Simão et al., 2010; Spinetti et al., 2010). According to Simão et al. (2012), even considering acute responses or chronic adaptations, exercise order should be prioritized so that the exercises that best address individual needs and training objectives are performed first.

Recently, Simão et al. (2013) showed that several acute studies had examined the effect of exercise order (Chaves et al., 2013; Figueiredo et al., 2011; Miranda et al., 2010; Bellezza et al., 2009; Farinatti et al., 2009; Gentil et al., 2007; Simão et al., 2007; Spreuwenberg et al., 2006; Simão et al., 2005; Sforzo and Touey, 1996), but none of those studies investigated the effect of exercise order on hormonal responses to an exercise session. The volume completed during an RT session has been shown to vary with exercise order, and the magnitude of acute hormonal responses can vary in a similar fashion. Therefore, Simão et al. (2013) examined the acute hormonal responses to an upper-body RT session performed in opposite sequences (larger to smaller vs. smaller to larger muscle-group exercises). The main finding of this study was that exercise order affected the endocrine response to an upper-body resistance-exercise session.

The relevance of some circulating hormones for muscle adaptations to RT is highlighted by the findings that suppression of circulating testosterone concentrations prevents RT-induced hypertrophy in healthy men (Kvorning et al., 2006). Furthermore, Hansen et al. (2001) showed that strength increased more when exercise sessions included an acute elevation of anabolic hormones. Therefore, an exercise-order effect on the acute hormonal response to RT sessions could help to explain the differences in strength and hypertrophy found when certain exercises are placed first or last in sessions. In addition, because of the lack of studies on muscle group exercise order and hormonal responses to RT sessions, this study evaluated and compared the acute endocrine response of total testosterone (TTE), free testosterone (FTE) and cortisol (CO) during RT sessions performed following two different exercise sequences based on the muscle groups engaged. We hypothesized that different muscle group exercise order is able to alter the acute endocrine responses after a single RT session.

## Material and Methods

### Subjects

Eight healthy males with the mean of age of 28.8 ± 6.4 years were selected for the study. The exclusion criteria were: bone, muscle or joint injuries; cardiovascular disease; no organized athletic activity; use of pharmacological drugs; consumption of nutritional supplements; and engagement in strength training for at least six months that consisted of at least three training sessions per week. The subjects recruited were experienced in resistance training (Table 1). The sample size was chosen using the L * Power 3.1 software. Based on an a priori analysis, we adopted a power of 0.80, α = 0.05, correlation coefficient of 0.5, correction nonsphericity of 1 and effect size of 0.25, as suggested by Beck (2013). This analysis of the statistical power was performed to reduce the probability of type II error and to determine the minimum number of participants required for this investigation. We found that the sample size was sufficient to provide more than 80% statistical power.

### Procedures

1^st^ visit - All participants signed consent forms in accordance to the Declaration of Helsinki after full disclosure of the study methodology and organization. Next, each responded to the PAR-Q (Physical Activity Readiness Questionnaire) and had his body composition measured by the skinfold test and body mass index (BMI). This research project was approved by the ethics committee of the Federal University of Rio de Janeiro (UFRJ), RJ, Brazil, protocol number 43/2011.
2^nd^ Visit - The subjects returned to the laboratory to give blood samples for analysis of TTE, FTE and CO concentration after a supervised period of 30 min at rest.
3^rd^ Visit - The loads for the specific exercises were determined by a trial and error method for each participant, consisting of performing the exercises in the order proposed for the large to small group exercise (LG-SM), which assessed the correct load to perform the 10-repetition maximum test (10-RM) during the session.
4^th^ Visit – After 48 hours, the subjects performed the 10-RM again (re-test) in the LG-SM order. The results demonstrated an excellent intraclass correlation coefficient, between 0.90 and 0.99, for the bench press (BP), lat pulldown (LP), barbell shoulder press (BSP), triceps pushdown (TP) and barbell cut (BC).
5^th^ Visit - The subjects were submitted to the LG-SM exercise order and, after the last repetition of the last exercise, their blood samples were collected to measure the levels of TTE, FTE and CO for the same exercise order.
6^th^ Visit - The loads for each exercise were again determined through a trial and error method in the order of small to large muscle group exercise (SM-LG), which determined a correct load to perform 10-RM for this exercise order.
7^th^ Visit - After 48 hours, the subjects performed the 10-RM in the SM-LG exercise order (re-test). The results again showed an excellent intraclass correlation coefficient, between 0.90 and 0.99, for BC, TP, BSP, LA and BP.
8^th^ Visit - The subjects were submitted to the SM-LG exercise order. Immediately after the last repetition of the last exercise, the blood samples were collected to measure the levels of TTE, FTE and CO for the exercise order.

All visits occurred in the morning between 8–10 am with an interval between the visits of 48 hours. The participants were advised to maintain their normal meals and activities. Body mass was measured on a platform scale (Filizola), which was accurate to 0.1 kg. Body height was measured on a stadiometer (Cardiomed), with accuracy of 0.1 cm. All individuals were measured barefoot and wearing a swimsuit. The BMI was determined by the ratio of body mass to body height squared [body mass in kilograms (kg) and body height in meters (m)]. To evaluate body composition, calipers (Cescorf) were used and the indexes were calculated by the body density equation proposed by Jackson and Pollock (1978).

All participants in this study were experienced practitioners of RT and all exercises performed were familiar for them. To minimize errors during the exercises, a strategy proposed by Simão et al. (2010) and Senna et al. (2009) was implemented, whereby the participants received standardized instructions that took into account the procedures before each test and standardized instructions of techniques used during the exercises. Verbal encouragement and free weights were used during the exercises and the weight of the equipment was confirmed on a platform scale.

To determine the load of each exercise, all participants had a maximum of five trials at each exercise with rest intervals ranging from 3 to 5 minutes. After load assessment, a 10 min rest period was allowed between exercises. The following standardized techniques were applied for each exercise:

BP - The subject lay down on a horizontal bench with his buttocks in contact with the bench and feet flat against the ground. The bar was held in the hands in a pronation position with a distance of more than shoulder width. To perform the exercise, the bar was removed from the support with the help of an assistant and the bar was lowered to a 90° angle between the arm and forearm (eccentric phase). Next the bar was raised to complete a full extension of the elbows (concentric phase).
LP - The subject began with his elbows fully extended and the pull was performed with the hands pronated until the bar touched lightly on the collarbone (concentric phase). Next, the weight was returned to the starting position to complete the full extension (eccentric phase).
BSP - The subject sat on a horizontal bench and the exercise began with the bar resting on the upper chest, where the elbows had full extension (concentric phase). Next, the elbows were flexed until they leaned slightly into the upper chest (eccentric phase).
TP - The subject began by holding the bar with pronated hands and the elbows maintained at an angle of 90° to the body. The exercise started with a full elbow extension (concentric phase) and ended with a flex of the elbow back to the angle of 90 ° (eccentric phase).
BC - The subject held the bar with pronated hands and maintained his elbows very close to the body in full elbow extension. The movement began with flexing of the elbows as much as possible (concentric phase), followed by full extension back to the starting position (eccentric phase).

The exercises were performed following two different exercise sequences. Sequence LG-SM began with exercises of the large muscle groups and progressed to exercises with the small muscle groups by implementing the sequence of BP to LP, BSP, TP and BC. Sequence SM-LG began with the small muscle groups and progressed to the large muscle groups by the sequence BC to TP, BSP, LP and BP. For all exercises in both sequences, there were three sets of 10 repetitions, with rest intervals of 2 min between sets and 3 min between exercises.

Venous blood samples (5 mL) were collected using sterile surgical gloves, needles and syringes by an experienced nurse from the superficial veins of the arm (venipuncture). Collection occurred at rest and at the end of the last set of the last exercise of the sequence evaluated. The blood was stored in plastic tubes and transported in an insulated box to the laboratory where the serum was separated and stored at −4 ºC in a freezer until analysis. To evaluate changes in hormone plasma levels, the chemiluminescent assay (Oliveira et al., 2008) was used and for TTE, a radioimmunoassay for CO was carried out as well (Riad-Fahmy et al., 1979). FTE was calculated via the Sodergard method (Sodergard et al., 1982).

### Statistical Analyses

All data are presented as mean ± standard deviation. First, normality and homoscedasticity were assessed by the Shapiro-Will test and the Bartlett criterion. All variables exhibited homoscedasticity and normal distribution. Oneway ANOVA with repeated measures was used to compare the differences between the pre-experimental and post-experimental situations as well as the differences between groups. Specific differences were determined using the Tukey’s post hoc test. An alpha level of p < 0.05 was considered statistically significant for all the comparisons. Additionally, to determine the magnitude of the results, effect sizes (ESs; the difference between pretest and posttest results divided by the pretest SD) were calculated for TTE, FTE and CO responses for both exercise sequences, and the scale proposed by Rhea (2004) was used to determine the magnitude of the ES.

## Results

The analysis of TTE showed significant differences (p < 0.05) for the overall effect between mean levels of TTE at rest when compared with the levels after the LG-SM and SM-LG routines (Figure 2). The TTE levels increased from 354.1 ng/dL at rest to 404 ng/dL at the end of the LG-SM routine and 411.3 ng/dL after the SM-LG. There was no significant difference between the values for the two sequences (p > 0.05).

**Figure 1:**
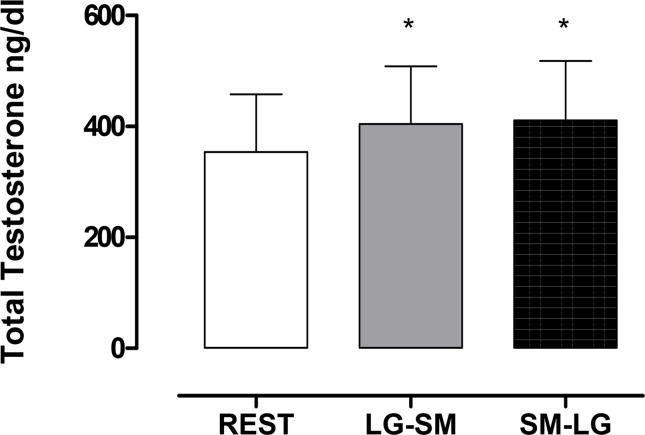
Comparisons of mean total testosterone (TTE) levels between rest, LG-SM and SM-LG protocols. * represents significant differences to baseline (rest vs. LG-SM and rest vs. SM-LG; p< 0.05)

**Figure 2:**
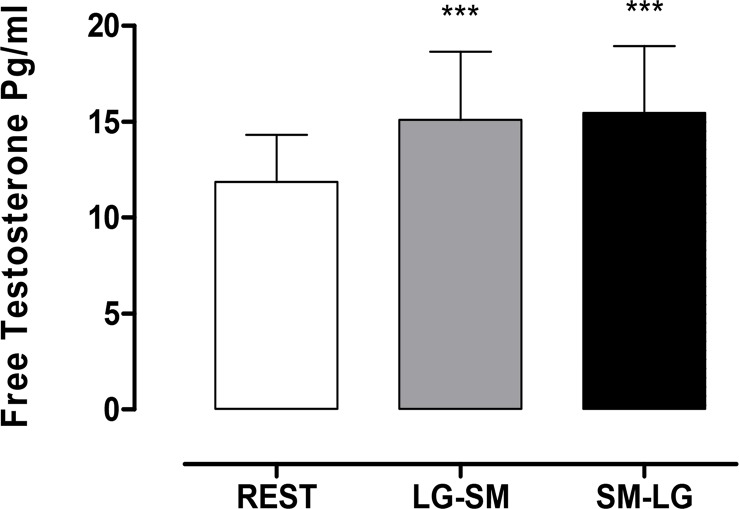
Comparisons of mean free testosterone (FTE) levels between rest, LG-SM and SM-LG protocols. *** represents significant differences to baseline (rest vs. LG-SM and rest vs. SM-LG; p< 0.0001)

The analysis of FTE showed a significant difference (p < 0.0001) for the overall effect between mean levels of FTE at rest when compared with the levels after sessions in both exercise orders (Figure 1). The FTE values increased from 11.85 pg/ml at rest to 15.10 pg/ml after performing the LG-SM routine and 15.46 pg/ml after the SM-LG. No significant differences were observed between the values for the two sequences proposed in our protocol (p > 0.05).

**Figure 3:**
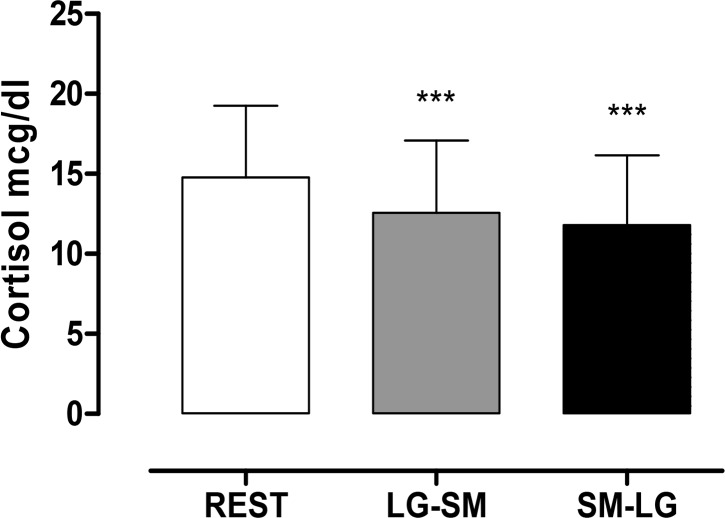
Comparisons of mean cortisol (CO) levels between rest, LG-SM and SM-LG protocols. *** represents significant differences to baseline (rest vs. SM-LG and rest vs. SM-LG; p< 0.0001)

The analysis of CO showed a significant difference (p < 0.0001) in the overall effect of the RT between mean levels of CO at rest when compared with the levels after the LG-SM and SM-LG routines (Figure 3). A decrease was observed from the resting value of 14.76 mcg/dL to 12:56 mcg/dL after LG-SM and 11.8 mcg/dL after SM-LG. There was no significant difference between the values for the two sequences (p > 0.05).

The effect-size analysis demonstrated moderate magnitude in the changes of TTE, and small magnitudes for FTE and CO in both exercise sequences.

## Discussion

In a recent study by Simão et al. (2013), 20 men completed 2 sessions (3 sets; 70% one-repetition maximum; 2 minutes of passive rest between sets) of the same exercises in opposite sequences (larger to smaller vs. smaller to larger muscle groups). Total testosterone (TTE), free testosterone (FTE), testosterone/cortisol ratio (TE/CO), sex-hormone-binding globulin (SHBG), growth hormone (GH), and cortisol (CO) concentrations were measured before and immediately after each sequence. The results indicated that GH concentration increased after both sessions, but the increase was significantly greater after the sequence in which larger muscle-group exercises were performed before the smaller muscle-group exercises. No differences were observed between sessions for TTE, FTE, SHBG, CO, or the TE/CO ratio at baseline or immediately after resistance exercise. These results indicate that performing larger muscle-group exercises first during an upper-body resistance-exercise session leads to a significantly greater GH response.

The main finding of this study is that exercise order does not affect the endocrine response of TTE, FTE and CO following an upper-body resistance-exercise session. These results indicate that from a practical standpoint, both sessions transiently altered the hormonal milieu in favor of muscle tissue building. Our results are in accordance with Simão et al. (2013), who did not find any significant difference between exercise order (LG-SM and SM-LG) for TTE, FTE, SHBG, CO, or the TE/CO ratio immediately after resistance exercise. In addition, our effect-size data demonstrated that differences in TTE, FTE and CO were not evident based on exercise order (LG-SM and SM-LG). In Simão et al.’s (2013) study, only the GH concentration showed an increase from LG-SM in the RT session. In our study, we did not measure the GH concentration.

An important factor should be noted in relation to acute hormonal responses from rest to the end of exercise for both LG-SM and SM-LG, because the exercises were performed based on the individual 10-RM load test. The evaluators provided no help during exercise movement execution. In a creative approach, Ahtiainen et al. (2003) submitted 16 subjects to two different protocols: a repetition maximum (RM) protocol, in which participants performed the exercises through 12-RM, and a second protocol of forced repetitions (FR), in which after the 12-RM exercise, a further 15% of the conventional load was added. The subjects were assisted by evaluators in order to conduct 3–5 more repetitions, which led to higher levels of responses in TTE and FTE from FR. Leite et al. (2011) assessed the levels of GH, CO and TTE after two intensities of different exercises. A significantly greater increase was measured in the levels of CO in sets that were performed with 12-RM. In contrast, an increase in the TE/CO ratio was observed after a 6-RM set was performed. With regard to GH, an increase occurred also after a 12-RM set. Our findings are consistent with these results because we found increased testosterone and reduced CO. This is different than Smilios et al. (2003), who examined the effects of the number of sets (2, 4 and 6) on acute changes in TTE, CO and GH after three different protocols: (1) strength, (2) muscle hypertrophy and (3) resisted force. They reported that the number of sets did not affect the concentrations of CO and TTE in any of the three protocols.

One limitation of our study was that all subjects had considerable experience in strength training, but none of them had engaged in a training program starting with smaller muscle groups. This factor may have contributed to the differences in our results, namely that we did not find a significant difference in concentrations of TTE, FTE and CO between the LG-SM and SM-LG exercise protocols, which leads us to believe that these responses are associated with individual performance in each type of training, possibly affecting acute hormonal responses. Such hypothesis was tested by Tremblay et al. (2003). The researchers recruited 23 healthy men who carried out resistance training (n = 7), endurance training (n = 8) or remained sedentary (n = 7). They performed measurements after the aerobic training or resistance training sessions and concluded that the hormonal profile was more dependent on the type and intensity of exercise than on exercise volume. They also observed that the strength training practitioners had a higher endocrine response to the exercises.

Linnamo et al. (2005) examined the acute hormonal responses in men and women within three different strength training protocols: (1) submaximal protocol, (2) maximum strength training protocol and (3) maximum explosive strength protocol. Their results indicated that during the maximum strength training protocol there were significant increases in TTE responses only in men while GH had a significant rise for both women and men. In comparison to our study, their participants only performed three exercises: sit-up, bench press and leg extension. Each was executed for 10-RM in 5 sets and with a 2 min rest interval between the sit-up and bench press and a 12 min rest interval before starting the leg extension exercise. In our study, we used a 2-min rest interval between sets and a 3-min rest interval between the exercises. We believe that this variation of intervals may have influenced the hormonal responses in the present study. Currently, no published studies have measured hormone responses after different sequences of exercises during resistance training. This study can be the first step for future research on how to evaluate the influence of exercise order on acute endocrine responses.

## Conclusions

Regarding the order of the exercises and its ability to alter the responses of TTE, FTE and CO, we found increased levels of anabolic hormones TTE and FTE only between post-exercise (LG-SM or SM-LG) and pre-exercise (resting) conditions. Thus, the development and implementation of exercises based on muscle size is not supported based on hormonal responses. According to this reasoning, acute hormonal responses do not change as a function of the order of exercises based on muscle size. This suggests that the order of exercises should be based on the training priority principle.

## Figures and Tables

**Table 1:** Morphological characteristics of the participants

**Subjects (n= 8)**	**Mean±SD**	**Min-Max**	**CV (%)**
Age (years)	28.8±4.6	21–36	15.9
Body mass (kg)	87.0±15.5	72–120	17.8
Body height (cm)	181.0±7.6	173–198	4.22
BMI (kg · m^−2^)	26.5±4.0	22.5–35.8	15.22
Body Fat (%)	10.0±3.9	5.6–15.8	39.2
Experience (years)	2.0±0.7	0.5–2.0	60.4

Standard Deviation (SD); Body Mass Index (BMI); Minimum-Maximum (Min-Max); Coefficient of Variation (CV)

**Table 2:** Effect size and magnitude of change in acute hormonal responses

	**LG-SM**	**SM-LG**
ES (magnitude) of TTE	−1.18 (Moderate)	−1.24 (Moderate)
ES (magnitude of FTE	0.55 (Small)	−0.62 (Small)
ES (magnitude) of CO	0.60 (Small)	0.63 (Small)

Effect Size (ES); Total Testosterone (TTE); Free Testosterone (FTE); Cortisol (CO); Order of large to small group exercise (LG-SM); Order of small to large group exercise (SM-LG)
